# Osteomyelitis is associated with increased anti-inflammatory response and immune exhaustion

**DOI:** 10.3389/fimmu.2024.1396592

**Published:** 2024-04-26

**Authors:** Jayagopi Surendar, Roslind K. Hackenberg, Fabio Schmitt-Sánchez, Robert Ossendorff, Kristian Welle, Birgit Stoffel-Wagner, Peter T. Sage, Christof Burger, Dieter C. Wirtz, Andreas C. Strauss, Frank A. Schildberg

**Affiliations:** ^1^ Department of Orthopedics and Trauma Surgery, University Hospital Bonn, Bonn, Germany; ^2^ Department of Hand, Plastic and Reconstructive Surgery, Burn Center, BG Trauma Center Ludwigshafen, University of Heidelberg, Ludwigshafen, Germany; ^3^ Institute of Clinical Chemistry and Clinical Pharmacology, University Hospital Bonn, Bonn, Germany; ^4^ Transplantation Research Center, Renal Division, Brigham and Women’s Hospital, Harvard Medical School, Boston, MA, United States

**Keywords:** osteomyelitis, infection, inflammation, immune exhaustion, T follicular helper cells, musculoskeletal immunology

## Abstract

**Introduction:**

Osteomyelitis (OMS) is a bone infection causing bone pain and severe complications. A balanced immune response is critical to eradicate infection without harming the host, yet pathogens manipulate immunity to establish a chronic infection. Understanding OMS-driven inflammation is essential for disease management, but comprehensive data on immune profiles and immune cell activation during OMS are lacking.

**Methods:**

Using high-dimensional flow cytometry, we investigated the detailed innate and adaptive systemic immune cell populations in OMS and age- and sex-matched controls.

**Results:**

Our study revealed that OMS is associated with increased levels of immune regulatory cells, namely T regulatory cells, B regulatory cells, and T follicular regulatory cells. In addition, the expression of immune activation markers HLA-DR and CD86 was decreased in OMS, while the expression of immune exhaustion markers TIM-3, PD-1, PD-L1, and VISTA was increased. Members of the T follicular helper (Tfh) cell family as well as classical and typical memory B cells were significantly increased in OMS individuals. We also found a strong correlation between memory B cells and Tfh cells.

**Discussion:**

We conclude that OMS skews the host immune system towards the immunomodulatory arm and that the Tfh memory B cell axis is evident in OMS. Therefore, immune-directed therapies may be a promising alternative for eradication and recurrence of infection in OMS, particularly in individuals and areas where antibiotic resistance is a major concern.

## Introduction

Osteomyelitis (OMS) is one of the oldest diseases known to humankind (1). It is inflammation and destruction of bone caused by bacteria, mycobacteria, or fungi characterized by bone pain and tenderness. Despite the advent of novel treatment strategies, it still poses a serious threat to humanity in the twenty-first century. The incidence of OMS is increasing in industrialized countries (2). Between 2008 and 2018, the prevalence of OMS in Germany increased by 10.44% (3). The cause of this upward trend is still unknown.

Traditionally, OMS is categorized into two types: hematogenous OMS and traumatic OMS (4). Hematogenous disease is more common in children under the age of 16, owing primarily to bacteremia (5). Traumatic OMS may develop as a result of surgery or fractures. Here, the skin’s natural barrier is broken, allowing harmful pathogens to penetrate the incision and infect bone tissue (6). In reality, due to the possibility of intraoperative bacterial translocation from the skin to the surgical site, all orthopedic surgeries are susceptible to OMS. *Staphylococcus aureus* (*S. aureus*) is the pathogen responsible for 30% to 60% of OMS occurrences in people (7), and staphylococci as a group are responsible for about 75% of cases in western countries (8). In other regions of the world, species like *Mycobacterium tuberculosis* and *Salmonella typhi* are more prevalent (6).

Osteoimmunology is a field that studies the interaction of immune cells with bones. Numerous studies have demonstrated that immune cells and bone cells namely osteoclasts, osteoblasts, and chondrocytes can interact, either directly or indirectly through soluble substances like cytokines and chemokines (9). The contribution of the immune system to the development of acute and chronic OMS has been studied using animal models. Immunomodulatory (M2) macrophages, which have poor phagocytic function, infiltrate around the bacteria in acute OMS (4). Bacterial infections influence the adaptive immune system in chronic OMS. During OMS, T cells in porcine are polarized towards T helper (Th)1 and Th17 cells; the cytokines produced by these cells are positive regulators of osteoclasts that promote bone loss (10). Furthermore, *S. aureus* has evasive strategies to circumvent B cell effector actions (11). Immune cells targeted therapy may be advantageous as antibiotic resistance becomes a significant problem in the treatment of OMS. Hence, it is important to conduct research to investigate the levels, and activation status of conventional and non-conventional innate and adaptive immune cells in humans.

Checkpoint molecules are regulators of immune cells; some are activators, and some are inhibitory molecules. These molecules are important to keep the immune system in balance. Since the last decade, checkpoint molecules have been investigated in various malignancies (12) and autoimmune diseases as potential immunotherapy targets (13). Among the various candidates, programmed cell death protein 1 (PD-1), cytotoxic T-lymphocyte-associated protein 4 (CTLA-4), and T-cell immunoglobulin and mucin-domain containing-3 (TIM-3) are extensively studied checkpoint molecules in various diseases. The impact of checkpoint molecules has been studied in other bone disorders such as rheumatoid arthritis (14) and osteoporosis (15), but reports on OMS are still scarce. In OMS, Wang et al. found higher percentages of CD4^+^ and CD8^+^ T cells that express the negative checkpoint molecule LAG-3^+^ (16). Checkpoint molecules also regulate other immune cells like monocytes, B cells, NK cells, and dendritic cells (DCs). Investigating the levels of various checkpoint molecules on diverse immune cells in OMS is crucial. This would eventually allow researchers to investigate the effects of specific checkpoint molecules on immune cells.

The gold standard method for diagnosing OMS is microbiologic and histopathologic assessment of bone samples, which can confirm infection and isolate pathogens. However, the diagnostic workup is time consuming and false negative results still occur. It, therefore, is important to identify the OMS subjects in a rapid and secure manner. Because once a biofilm has formed, eradication of infection without surgical treatment is impossible, and susceptibility to antibiotic treatments is considerably diminished (4). In a chronic implant-associated OMS mouse model, *S. aureus* builds up detectable quantities of biofilm in 7 days, peaking at 14 days (17). So, we need more and safer diagnostic methods to confirm the disease. Systemic immune cells and their activation status could serve as useful biomarkers. Our group has performed a high-dimensional analysis of immune cell composition in periprosthetic joint infections and proposed myeloid-derived suppressor cells (MDSC) as a potential biomarker for periprosthetic joint infections (18). In line with our previous study, we wanted to enumerate the systemic immune cells in OMS. We used multiparametric immune profiling of peripheral mononuclear cells (PBMC) from the study individuals and evaluated the activation and exhaustion levels of these immune cells to find distinguishing features in OMS patients compared to their counterparts.

## Methods

### Study subjects

In this prospective monocenter pilot study in a clinic of maximum medical care, all patients with a minimum age of 18 years and a suspected OMS of the limbs were included between January 2020 and March 2021. All patients included were willing to participate in the study and gave their written informed consent. The study was approved by the local ethics committee of the University Hospital Bonn (local review board number 277/19) and performed in accordance with the ethical standards of the institutional and national research committees and the 1964 Helsinki declaration and its later amendments.

In total, 25 study subjects were registered in the study. Among them, 9 individuals had chronic OMS and 16 were controls. We measured biochemical and clinical parameters, including sodium, potassium, calcium, creatinine, urea, and C-reactive protein (CRP), as well as hematological parameters, including leucocytes, erythrocytes, hemoglobin, hematocrit, mean corpuscular volume (MCV), mean corpuscular hemoglobin (MCH), mean corpuscular hemoglobin concentration (MCHC) with the aid of an autoanalyzer. Coagulation markers like the QUICK coagulation value, international normalized ratio (INR), and activated partial thromboplastin time (aPTT) were also measured using an autoanalyzer. OMS status was determined by blood CRP and leucocytes count (WBC), magnet resonance imaging (MRI), microbiological analysis, and histopathology samples collected from representative areas after a first debridement, as published previously (19).

### Sample preparation

PBMC were isolated using sepmate tubes according to the manufacturer’s instructions. In brief, blood from an EDTA vacutainer was diluted in an equal volume of PBS. Diluted blood was overlayered on Ficoll in sepmate tubes and spun at 1200 g for 10 minutes. The PBMC were washed and counted. Cells were cryopreserved in 10% DMSO in cryovials and initially stored at -80°C for 24 hours before being transferred to -150°C until use.

### Flow cytometry

The antibodies used in the study were from Biolegend, BD Biosciences, and R&D systems. To prepare the cells for staining, they were thawed, washed with buffer, and suspended in FACS buffer. 3 x 10^5^ – 5 x 10^5^ cells were used for staining. Non-specific antibody staining was prevented by using Fc block. For analysis, doublets were excluded by FSC-A vs FSC-H and FSC-W vs SSC-A. Live cells alone were used for the analysis by excluding Zombie Aqua dye positive cells.

For T cell phenotyping, antibodies against CD3-BV711, CD4-BUV496, CD8-BV605, CD95-BV421, CCR7-Percpcy5.5, PD1-PE, CD57-PEcy7, CD25-BUV737, CD45RA-APCcy7, CD127-BV650, HLADR-BV785, CD38-FITC, CD28-PE-Dazzle, CD27-BUV805 were used. T cells were gated with the aid of CD3, followed by CD4 and CD8. Regulatory T Cells (Tregs) were identified as CD4^+^CD25^+^CD127^-^ cells. CD4^+^ and CD8^+^ T cells were classified further as follows: naive T cells were classified as CD45RA^+^CCR7^+^, central memory as CD45RA^-^CCR7^+^, terminally differentiated effector memory (TEMRA) as CD45RA^+^CCR7^-^, and effector T cells as CD45RA^-^CCR7^-^ (20). To classify stem cell-like memory T cells (Tscm), naive T cells were further gated as CD27^+^CD28^+^CD95^+^. Activated T cells were gated as HLA-DR^+^CD38^+^, and senescent T cells were classified as CD28^-^CD57^+^. Exhausted/Senescence T cells were classified as PD1^+^CD57^+^ (21). Furthermore, activated CD4^+^ and CD8^+^ cells were also enumerated with the aid of CD25^+^ expression.

T follicular helper cells (Tfh) and unconventional T cell phenotyping was carried out by the antibodies directed against CD3-BUV805, CD4-BUV496, CD8-BUV395, CD161-APCFire 750, TCRVα7.2-BV785, TCR γ/δ-BV421, TCR Vδ1-FITC, TCR Vδ2-BV605, CXCR5-PEDazzle 594, CD45RA-BV650, CXCR3-AF700, CD25-BV711, CCR6-PEcy7 and CD127-Percpcy5.5. Tfh cells were identified as CD4^+^CXCR5^+^CD45RA^-^ cells. Tfh cells were subdivided further into Tfh-Th1 (CXCR3^+^CCR6^-^) and Tfh-Tfh17 (CXCR3^-^CCR6^+^). T follicular regulatory (Tfr) were classified as CD4^+^CD25^+^CD127^-^CXCR5^+^ (22, 23). γδ T cells were identified as CD3^+^CD4^-^CD8^-^ TCR γ/δ^+^. Subclassification of γδ T cells was performed with TCR Vδ1^+^ as γδ1 T cells and TCR Vδ2 ^+^ as γδ2 T cells (24). MAIT cells were identified as CD3^+^ TCR γ/δ^-^ TCR Vα7.2^+^CD161^+^. Subclassification of MAIT cells was performed with CD4 as CD4^+^MAIT cells and with CD8 as CD8^+^MAIT cells (25, 26). Activated Tfh, γδ T cells and MAIT cells were measured using CD25^+^.

For B cell phenotyping, the following antibodies were used: CD3-BV711, CD19-BV785, IgD-BV605, IgG-BUV 737, IgM-PEcy7, IgA-APC, CD21-BV421, CD20-BUV 805, CD38-PE Dazzle 594, CD24-Percpcy5.5, CD27-FITC, and CD10-PE. Total B cells were gated as CD3^-^CD19^+^ and these cells were selected for B cell subset classification. Naive B cells were classified as CD21^+^CD27^-^, classical memory as CD21^+^CD27^+^, activated memory as CD21^-^CD27^+^ and atypical memory as CD21^-^CD27^-^ (27, 28). CD10 and IgM were used to classify immature transitional and mature B cells. Transitional B cells were classified as CD10^+^IgM^+^ and mature B cells as CD10^-^IgM^+^. Mature B cells were further classified using IgD and CD27 as IgD^+^CD27^+^ and IgD^-^CD27^+^. Marginal zone and IgD only memory cells were gated from IgD^+^CD27^+^ cells using CD27 and IgM. There were few IgD^+^CD27^+^-(IgD only) memory B cells that lack IgM, but the majority of IgD^+^CD27^+^ cells were marginal zone B cells that express high levels of IgM. Plasmablasts (CD20^-^CD27^high^) and memory B cells (CD20^+^CD27^+^) were defined from IgD^-^ B cells. Memory B cells were further divided into IgM^+^ (IgG^-^IgM^+^), IgG^+^ (IgG^+^IgM^-^), and IgA^+^ (IgG^-^IgM^-^IgA^+^) memory B cells (29). For regulatory B cells (Bregs), total B cells were gated into two Bregs subsets CD24^high^CD27^+^ and CD24^high^CD38^high^ (30).

For myeloid and natural killer (NK) cell phenotyping, two flow cytometry panels were used. CD45-BV650, Lin-APC, CD123-BV786, HLADR-APCcy7, CD141-BV605, CD11c-PEcy7, CD1c-PE Dazzle, CD14-BUV 805, CD3/CD19/CD20-BV605, CD33-PEcy7, CD11b-PE Dazzle, and HLADR-BV650 antibodies were used for the analysis. For monocyte identification, lymphocytes (CD3/CD19/CD20)^+^ and CD56^+^ cells were excluded. From the non-lymphocytes and CD56^-^ cells, classical monocytes (CD14^high^/CD16^-^), intermediate monocytes (CD14^high^/CD16^dim^), and non-classical monocytes (CD14^dim^/CD16^high^) were identified (31). Myeloid DCs (mDC) were classified as Lin^-^HLA-DR^+^ CD11c and plasmacytoid DCs (pDC) as Lin^-^HLA-DR^+^ CD123^+^. mDC were further subclassified into cDC1 (CD141^+^) and cDC2(CD1c) (32, 33). MDSC were identified as lymphocyte (CD3CD19/CD20)^-^CD16^-^CD56^-^HLADR^-^CD33^+^CD11b^+^ (34). eMDSC were identified as lymphocyte (CD3CD19/CD20)^-^CD14^-^CD16^-^CD56^-^HLADR^-^CD33^+^ (35). For the NK cell family, early NK cells were classified as lymphoytes^-^CD56^+^CD16^-^, mature NK cells as lymphocytes^-^ CD16^+^CD56^+^ and late NK cells as lymphoytes^-^CD56^-^CD16^+^ (36).

To measure mean fluorescence intensity (MFI) of activation and exhaustion molecules on the immune cells, the following antibodies were used: PD1-PE, TIM3-APC, TIM3-APCcy7, HLADR-BV650, PDL1-BV711, VISTA-FITC, CD86-BV785, galectin 9 (Gal-9)-percpcy5.5 and CD80-AF700.

Antibody-stained cells were acquired on a LSR Fortessa analyzer 10 flow cytometer with FACS DIVA software (BD Biosciences). Laser calibration was done in all the experiments using CS&T beads (BD Biosciences). FlowJo software was used for data analysis using FMO samples. Batched gates occasionally needed manual correction to account for tiny variations between samples because of the nature of the heterogeneity of the subjects.

### Statistical analysis

The central tendency was measured as mean +/- standard deviation. For continuous variables with a normal distribution, the student’s t test was used to compare groups, whereas the Mann-Whitney U test was used for continuous variables that did not follow a normal distribution. Chi-square test was used to compare groups for proportions. A Pearson correlation analysis was carried out to determine the relation of Tfh with activated memory and classical memory B cells. Graphpad Prism version 9.4.1 was used for analysis.

## Results

We first performed a detailed biochemical analysis of all blood samples from the study cohort. **Table 1** depicts the clinical and biochemical characteristics of the study subjects. Both the control and OMS subjects were age- and gender-matched. Sodium, potassium, calcium, creatinine, urea, and CRP levels were comparable between the groups. The hematological parameters leucocytes, and platelet levels were significantly higher in OMS subjects, but hemoglobin and hematocrit levels were significantly lower. However, other hematological parameters such as erythrocytes, MCV, MCH, MCHC, RDW, and MPV levels were not significantly altered between the groups. Coagulation parameters QUICK, and INR levels were significantly lower and higher in OMS groups compared to controls, respectively, but aPTT levels were not significantly different.

**Table 1:** Clinical characteristics of the study subjects.

Parameters	Controls (n=16)	OMS (n=9)	p
Age (years)	47.1 ± 15.6	55.7 ± 19.0	0.234
Gender (M/F) (n)	9/7	6/3	0.691
Sodium (mmol/l)	139.9 ± 1.5	138.9 ± 4.4	0.412
Potassium (mmol/l)	4.4 ± 0.3	4.5 ± 0.7	0.441
Calcium (mmol/l)	2.4 ± 0.1	2.0 ± 0.6	0.051
Creatinine (mg/dl)	0.9 ± 0.4	1.0 ± 0.3	0.980
Urea (mg/dl)	26.7 ± 6.7	31.3 ± 21.1	0.460
CRP (mg/l)	9.6 ± 13.1	51.2 ± 67.3	0.088
Hematology			
Leucocytes (G/l)	6.7 ± 2.5	9.2 ± 2.8	**0.032**
Erythrocytes (T/l)	4.8 ± 0.6	4.2 ± 0.8	0.057
Hemoglobin (g/dl)	14.2 ± 2.0	12.4 ± 2.1	**0.045**
Hematocrit (%)	42.3 ± 5.0	37 ± 6.5	**0.033**
MCV (fl)	88.1 ± 3.7	87.4 ± 4.1	0.673
MCH (pg)	29.6 ± 1.7	29.6 ± 1.8	0.924
MCHC (g/dl)	33.7 ± 1.4	33.7 ± 1.1	0.971
RDW (%)	13.6 ± 2.3	15.7 ± 3.4	0.070
Platelets (G/l)	254 ± 59	354 ± 78	**0.002**
MPV (fl)	10.3 ± 0.8	10.2 ± 0.9	0.672
Coagulation			
QUICK coagulation value (%)	105 ± 14	84 ± 14	**0.009**
International Normalized Ratio (INR)	0.98 ± 0.08	1.06 ± 0.07	**0.039**
aPTT (sec)	24.3 ± 1.7	26.6 ± 3.6	0.068

MCV, Mean corpuscular volume; MCH, Mean corpuscular hemoglobin; MCHC, Mean corpuscular hemoglobin concentration; RDW, Red blood cell distribution width; MPV, mean platelet volume; aPTT, activated partial thromboplastin time; p-values less than 0.05 are mentioned in bold..

**Figure 1:**
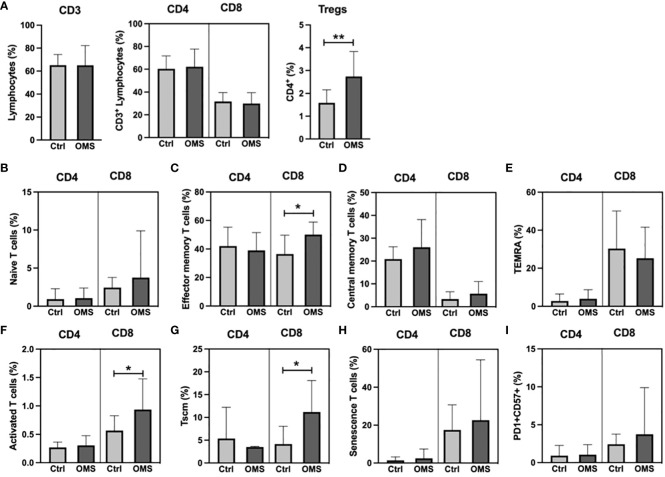
Elevated Treg frequency in OMS subjects. **(A)** T cell frequencies (CD3^+^, CD4^+^, CD8^+^, and Tregs). Frequencies of **(B)** naïve, **(C)** effector memory, **(D)** central memory, **(E)** TEMRA, **(F)** activated, **(G)** Tscm, **(H)** senescence, and **(I)** PD1^+^CD57^+^ CD4^+^ and CD8^+^ T cells in control and OMS subjects. The bars represent the mean ± SD. Statistical significance was determined by student t or Mann-Whitney U test and *p<0.05, **p<0.01.

To determine the influence of OMS infection on the systemic T cell profile, we enumerated the frequencies of various T cell subsets as shown in **Figure 1**. OMS subjects had significantly increased frequencies of Tregs but not of pan CD3^+^, CD4^+^, and CD8^+^ T cells, as depicted in **Figure 1A**. The frequencies of CD4^+^ naive T cells, effector memory T cells, central memory T cells, TEMRA, activated T cells, Tscm, senescent T cells, and PD-1^+^CD57^+^ were comparable between control and OMS subjects (**Figures 1B–I**). The frequencies of effector memory (**Figure 1C**), activated (**Figure 1F**), and Tscm (**Figure 1G**) CD8^+^ T cells were significantly increased in OMS subjects, but no significant differences in CD8^+^ naive T cells (**Figure 1B**), central memory T cells (**Figure 1D**), TEMRA (**Figure 1E**), senescence T cells (**Figure 1H**), and PD-1^+^CD57^+^ (**Figure 1I**) cells were found between controls and OMS subjects. In addition, CD25^+^CD4^+^ and CD25^+^CD8^+^ T cells were significantly increased in OMS subjects (**Supplementary Figure 1A**). Thus, OMS infection is associated with profound alterations in CD25^+^ activated T cells, circulating Tregs and specific CD8^+^ subsets.

**Figure 2:**
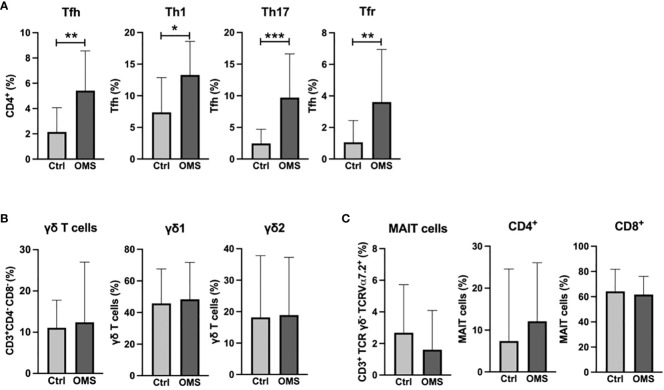
Elevated percentage of the Tfh family in the OMS group. **(A)** Frequencies of Tfh family (total Tfh, Tfh-Th1, Tfh-Th17 and Tfr). **(B)** Frequencies of γδ family (total γδ T cells, γδ1 T cells and γδ2 T cells). **(C)** Frequencies of MAIT cells (total MAIT, CD4^+^ MAIT cells and CD8^+^ MAIT cells) in control and OMS subjects. The bars represent the mean ± SD. Statistical significance was determined by student t or Mann-Whitney U test and *p<0.05, **p<0.01, ***p<0.001.

To elucidate the effect of OMS infection on systemic levels of specialized T cells and unconventional T cells, we determined the frequencies of Tfh cells and γδ T cells, and MAIT cells, respectively, as shown in **Figure 2**. OMS individuals exhibited significantly increased frequencies of total Tfh and Tfh subsets, namely, Tfh-Th1, Tfh-Th17, and Tfr, compared to controls, as depicted in **Figure 2A**. The frequencies of the γδ T cell family and the MAIT cell family, on the other hand, did not differ between OMS subjects and their counterparts, as shown in **Figures 2B, C**. OMS subjects showed marginally elevated levels of CD25^+^ γδ T cells (p=0.055) and significantly increased levels of CD25^+^ Tfh and CD25^+^ MAIT cells (**Supplementary Figures 1B, C**). Thus, OMS infection is associated with substantial alterations to the Tfh family and activation of Tfh and MAIT cells.

**Figure 3:**
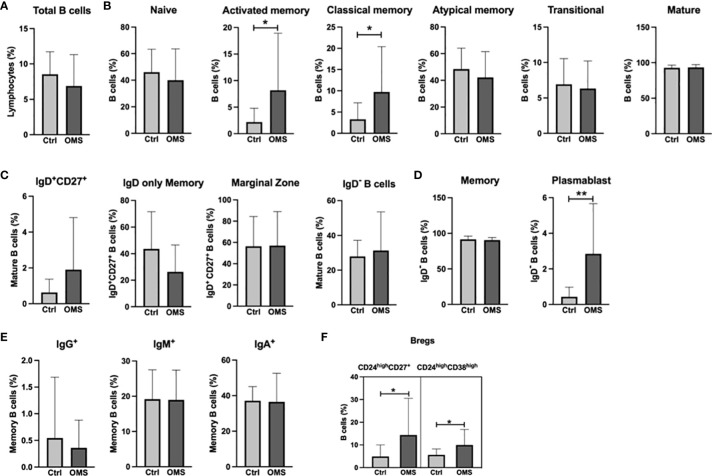
Increased frequencies of specific memory B cell subsets, plasmablasts, and Bregs in OMS subjects. Depicted are B cell family subsets in control and OMS subjects. **(A)** Frequencies of total B cells. **(B)** Frequencies of B cell subsets: naive B cells, activated memory, classical memory, atypical memory, transitional, and mature B cells. **(C)** Frequencies of mature B cell subsets: IgD^+^CD27^+^, IgD only memory cells, marginal zone B cells, and IgD^-^ B cells. **(D)** Frequencies of total memory B cells and plasmablasts. **(E)** Frequencies of memory B cell subsets (IgG^+^, IgM^+^, IgA^+^). **(F)** Frequencies of the Bregs subsets CD24^high^CD27^+^ and CD24^high^CD38^high^. The bars represent the mean ± SD. Statistical significance was determined by student t or Mann-Whitney U test and *p<0.05, **p<0.01.

To determine the effect of OMS infection on systemic levels of B cell subsets, we determined the various B cell subset frequencies in the study subjects as shown in **Figure 3**. The total B cell frequencies were not altered between the OMS and control groups, as shown in **Figure 3A**. OMS subjects exhibited increased frequencies of activated memory and classical memory B cell subsets but not naive, atypical, transitional, and mature B cells (**Figure 3B**). In addition, mature B cell subsets (**Figure 3C**) and memory B cells and their subsets (**Figures 3D, E**) were comparable between OMS and control groups. Plasmablast frequencies were significantly increased in OMS groups (**Figure 3D**). Interestingly, the frequencies of the Breg types CD24^high^CD27^+^ and CD24^high^CD38^high^ were significantly increased in the OMS group compared to controls (**Figure 3F**). Total Tfh frequencies were significantly correlated with activated memory (r = 0.558, p = 0.009) and classical memory (r = 0.0573, p = 0.006) B cells (data not shown). Therefore, OMS is associated with profound alterations in the frequency of specific B cell subsets, and Tfh cells showed a positive association with activated and classical memory B cells.

**Figure 4:**
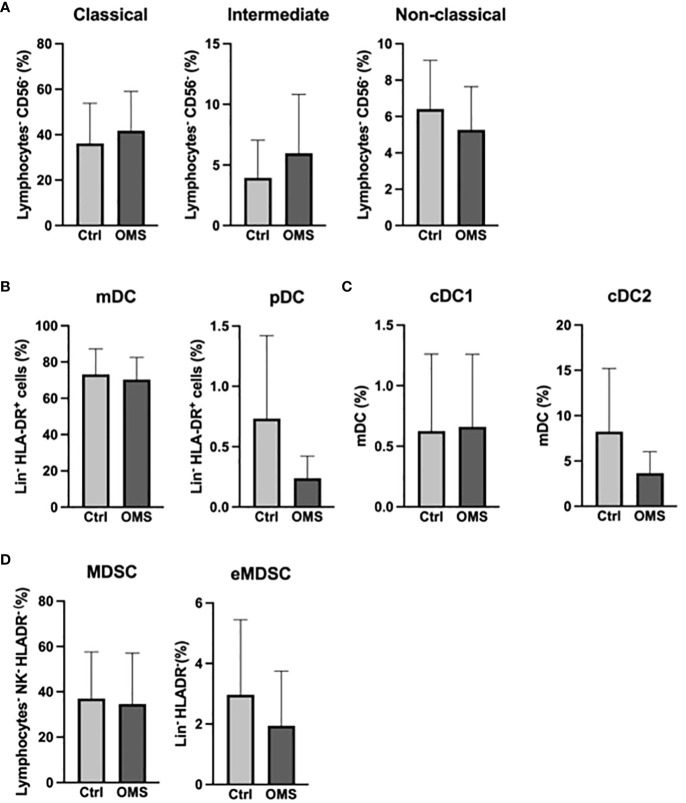
Unaltered levels of myeloid compartment. **(A)** Frequencies of the monocyte family (classical, intermediate, and non-classical monocytes). **(B)** Frequencies of DC subsets (mDC and pDC). **(C)** Frequencies of mDC subsets (cDC1 and cDC2). **(D)** Frequencies of MDSC and eMDSC in control and OMS subjects. The bars represent the mean ± SD. Statistical significance was determined by student t or Mann-Whitney U test.

To determine the effect of OMS infection on systemic levels of the myeloid cell pool, we enumerated the frequencies of various monocyte, DC, and MDSC subsets in the study subjects as shown in **Figure 4**. The frequencies of monocyte subsets were unaltered between OMS and control groups (**Figure 4A**). Similarly, frequencies of mDC and pDC were comparable between the groups (**Figure 4B**). As can be seen in **Figure 4C**, OMS subjects had similar levels of cDC1 and cDC2 compared to their counterparts. The frequencies of MDSC and eMDSC cells were comparable between the control and OMS groups, as shown in **Figure 4D**. Thus, OMS is not associated with systemic myeloid cell frequencies.

**Figure 5:**
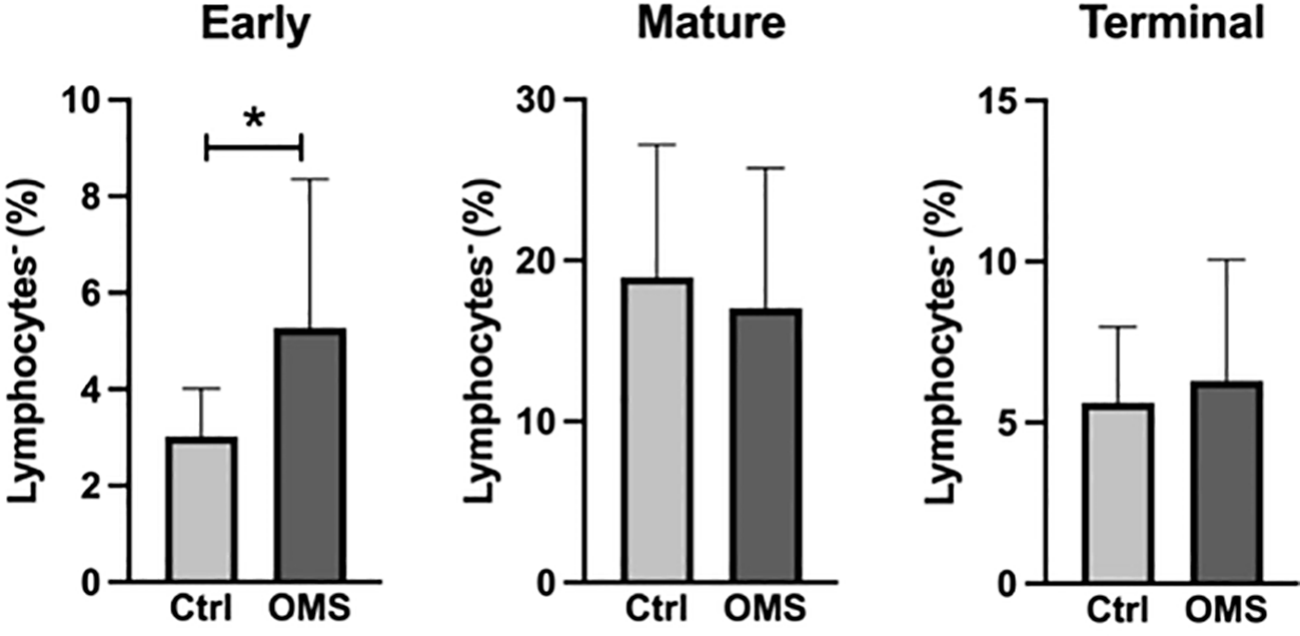
Increased frequencies of early NK cells. Frequencies of the NK cell family (early, mature, and terminal NK cells). The bars represent the mean ± SD. Statistical significance was determined by student t or Mann-Whitney U test and *p<0.05.

To determine the impact of OMS infection on the frequencies of NK cell subsets, we enumerated the frequencies of various NK cell subsets in the study groups, as shown in **Figure 5**. OMS subjects exhibited significantly increased frequencies of early NK cells but did not exhibit any significant differences in mature and terminal NK cell frequencies, as depicted in **Figure 5**. Thus, OMS is associated with augmented frequencies of early NK cells.

**Figure 6:**
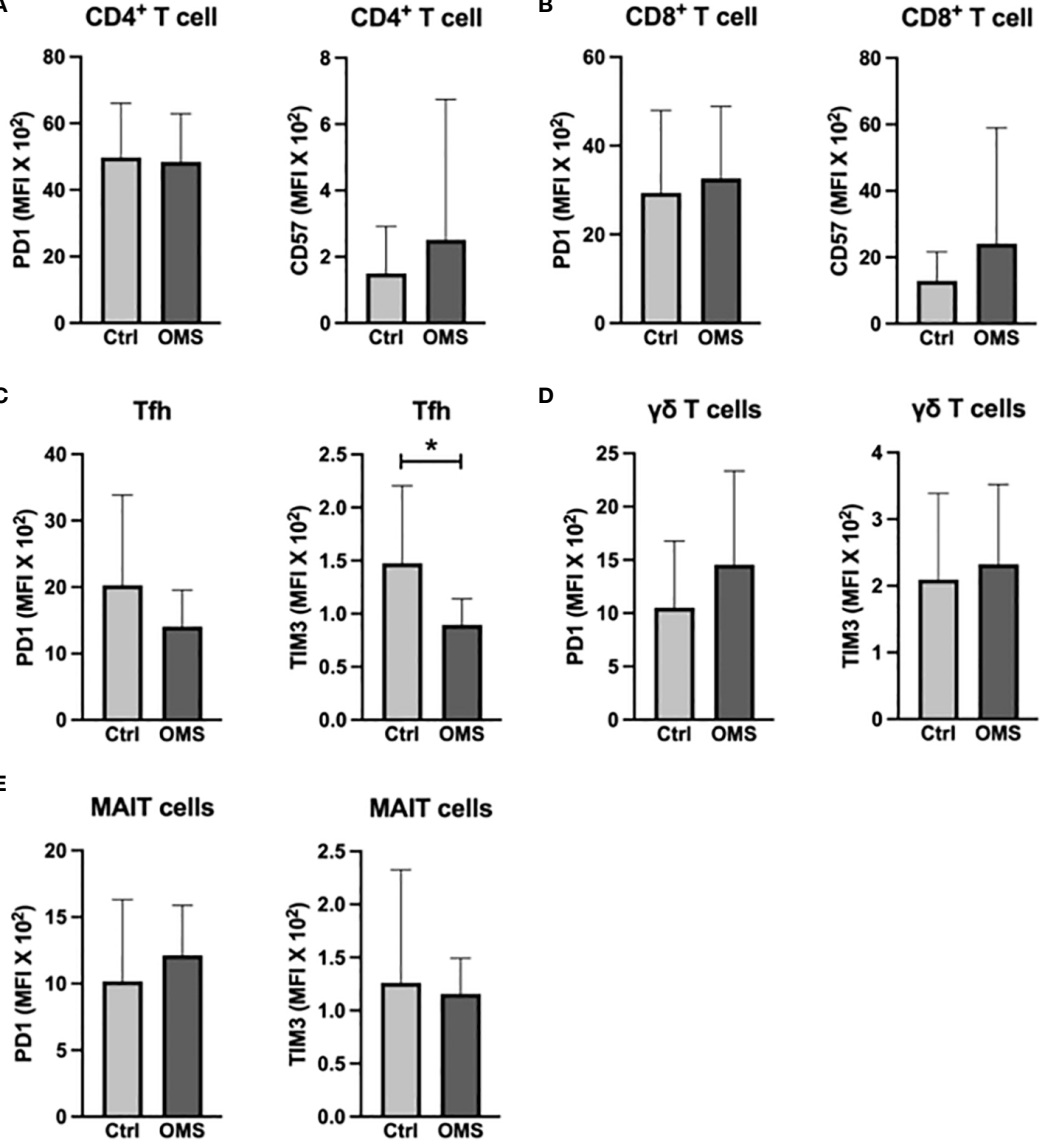
OMS decreases TIM3 expression on Tfh cells. MFI values of PD1 and CD57 **(A)** in CD4^+^ T cells and **(B)** in CD8^+^ T cells. MFI values of PD1 and TIM3 **(C)** in Tfh, **(D)** in γδ T cells, and in **(E)** MAIT cells. The bars represent the mean ± SD. Statistical significance was determined by student t or Mann-Whitney U test and *p<0.05.

To determine the influence of senescence and exhaustion molecule expression in conventional and unconventional T cells, we analyzed the MFI value of senescence and exhaustion molecules on T cells, Tfh, γδ T cells, and MAIT cells, as shown in **Figure 6**. OMS subjects did not exhibit any significant change in the MFI values of PD-1 and CD57 in CD4^+^ and CD8^+^ T cells compared to the control groups (**Figures 6A, B**). However, the value of TIM-3 in Tfh cells in OMS subjects was significantly lower than in controls but not the MFI value of PD-1 (**Figure 6C**). In addition, the MFI values of PD-1 and TIM-3 in γδ T cells and MAIT cells were comparable between controls and OMS subjects (**Figures 6D, E**). Thus, OMS infection is associated with reduced MFI of TIM-3 on Tfh cells.

**Figure 7:**
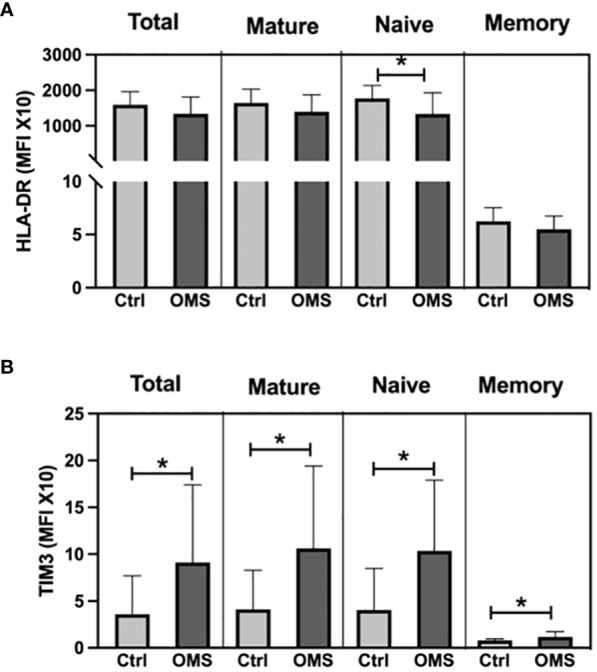
OMS infection increases the TIM3 expression on B cells. **(A)** MFI values of HLA-DR on B cell subsets (total, mature, naive and, memory B cells), **(B)** MFI values of TIM3 on B cell subsets. The bars represent the mean ± SD. Statistical significance was determined by student t or Mann-Whitney U test and *p<0.05.

To determine the effect of OMS on activation and exhaustion molecule expression on B cells and its subsets, we quantified the MFI of HLA-DR and TIM-3 in total, naive, mature, and memory B cells, as shown in **Figure 7**. When comparing OMS subjects to controls, the MFI value of HLA-DR was significantly lower in naive B cells, but not in total, mature, or memory B cells (**Figure 7A**). Interestingly, the OMS group showed an increased MFI value of TIM-3 in total B, naive, mature, and memory B cells (**Figure 7B**). Thus, OMS is associated with increased expression of exhaustion molecules on B cells and their subsets.

**Figure 8:**
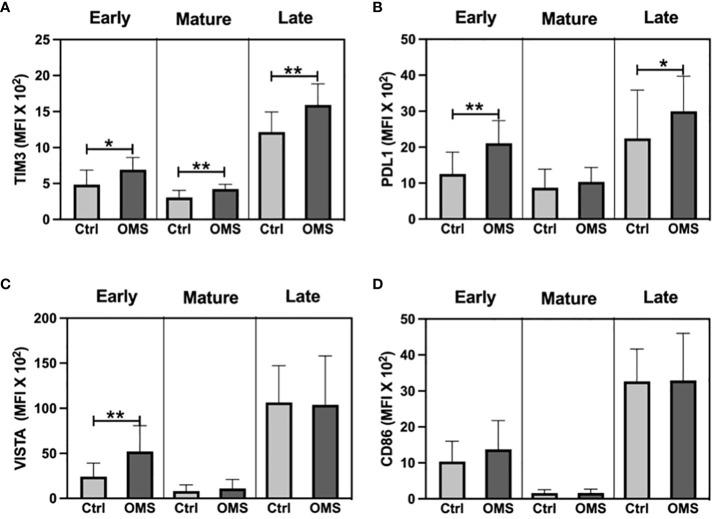
OMS infection increases exhaustion molecule expression on the NK cell family. MFI value of **(A)** TIM3, **(B)** PDL1, **(C)** VISTA, and **(D)** CD86 in NK cell subsets (early, mature, and late). The bars represent the mean ± SD. Statistical significance was determined by student t or Mann-Whitney U test and *p<0.05, **p<0.01.

To determine the effect of OMS on activation and exhaustion molecule expression on NK cell subsets, we measured the MFI of TIM-3, PD-L1, VISTA, and CD86 on different NK cell subsets as shown in **Figure 8**. The OMS group showed significantly increased MFI values of TIM-3 on early, mature, and late NK cells (**Figure 8A**). Similarly, OMS subjects had a significantly elevated MFI value of PD-L1 on early and late NK cells but not in mature NK cells (**Figure 8B**). In addition, the MFI value of VISTA was significantly increased in early NK cells in OMS subjects but not in mature and late NK cells (**Figure 8C**). The MFI value of the activation marker, CD86 was comparable between the groups in NK cell subsets (**Figure 8D**). Therefore, OMS is associated with an increased MFI value of exhaustion molecules on NK cell subsets.

**Figure 9:**
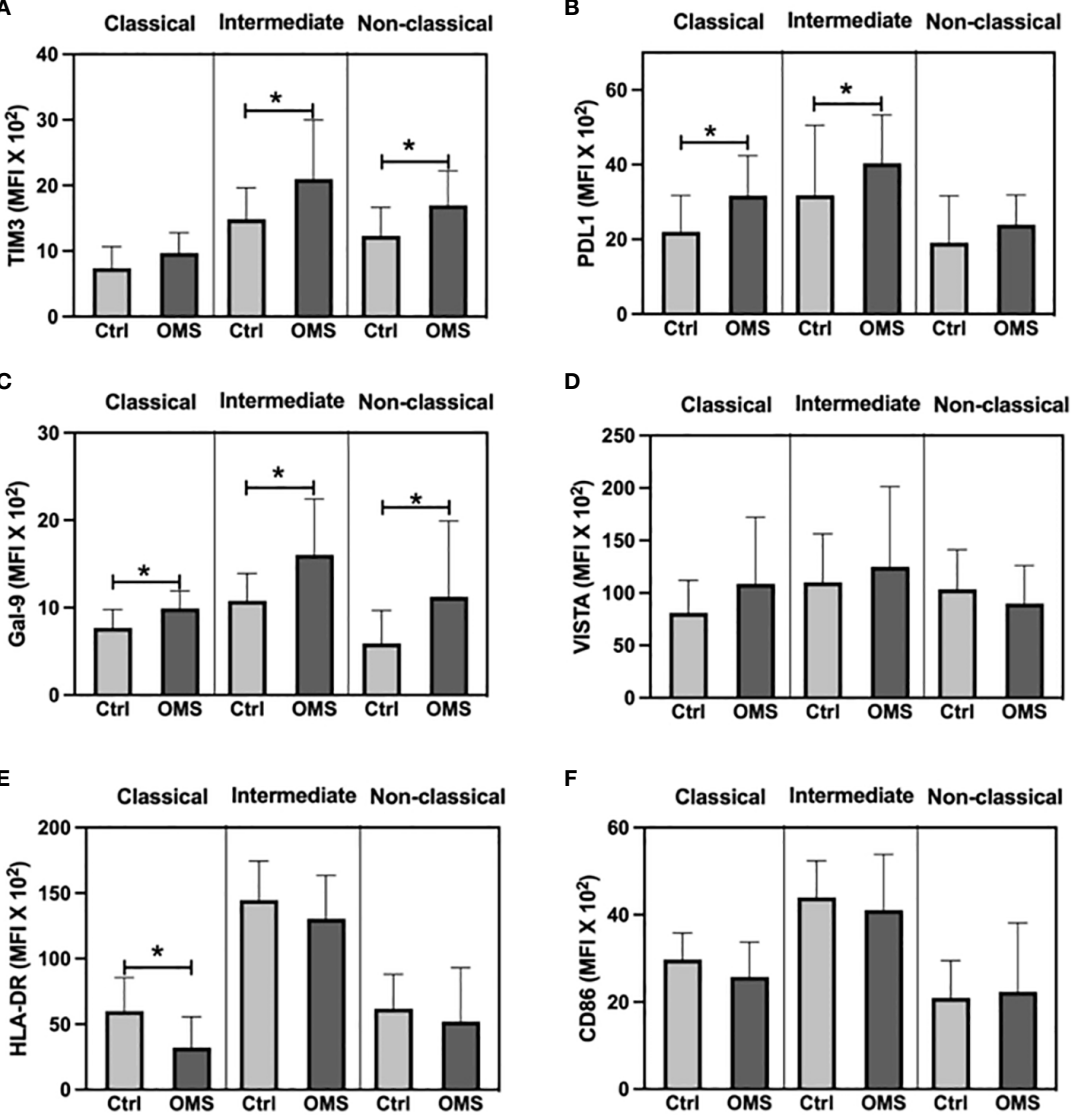
OMS infection increases exhaustion molecule expression on monocyte subsets. MFI value of **(A)** TIM3, **(B)** PDL1, **(C)** Gal-9, **(D)** VISTA, **(E)** HLA-DR, and **(F)** CD86 in monocyte subsets (classical, intermediate, and non-classical monocytes). Statistical significance was determined by student t or Mann-Whitney U test and *p<0.05.

To determine the impact of OMS on activation and exhaustion molecule expression on monocyte subsets, we measured the MFI values of TIM-3, PD-L1, Gal-9, VISTA, HLA-DR, and CD86 on monocyte subsets as shown in **Figure 9**. The OMS group showed increased MFI of TIM-3 in intermediate and non-classical monocytes (**Figure 9A**). The MFI value of PD-L1 was significantly elevated in the OMS group compared to controls in classical and intermediate monocytes but not in non-classical monocytes (**Figure 9B**). In addition, subjects with OMS had increased MFI of Gal-9 in classical, intermediate, and non-classical monocytes (**Figure 9C**). However, the MFI value of VISTA was comparable between the groups (**Figure 9D**). OMS individuals showed a reduced MFI value of HLA-DR in classical monocytes but no change in intermediate and non-classical monocytes (**Figure 9E**). Furthermore, the MFI value of CD86 was comparable between the groups (**Figure 9F**). Thus, OMS is associated with increased MFI of exhaustion molecules on monocyte subsets.

**Figure 10:**
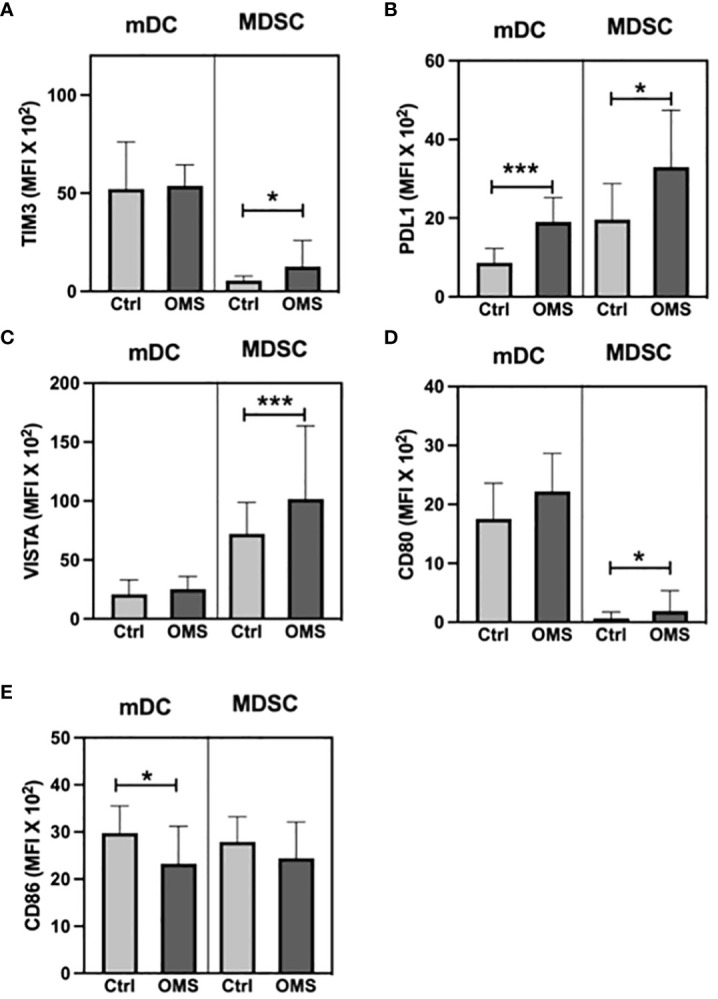
OMS infection increases exhaustion molecule expression on mDC and MDSC. MFI value of **(A)** TIM 3, **(B)** PDL1, **(C)** VISTA, **(D)** CD80, and **(E)** CD86 in mDC and MDSC. Statistical significance was determined by student t or Mann-Whitney U test and *p<0.05, ***p<0.001.

To determine the impact of OMS on activation and exhaustion molecule expression in mDC and MDSC, we enumerated the MFI values of TIM-3, PD-L1, VISTA, CD80, and CD86 in mDC and MDSC as shown in **Figure 10**. The MFI values of TIM-3 and VISTA was significantly increased in MDSC in the OMS group, but no change was observed in the mDC population (**Figures 10A, C**). Furthermore, OMS subjects exhibited increased MFI values for PD-L1 in both mDC and MDSC as depicted in **Figure 10B**. In addition, OMS subjects exhibited an increased MFI value of CD80, an activation marker, in MDSC but not in the mDC population (**Figure 10D**). In contrast, for another activation marker, CD86, the MFI value was significantly reduced in OMS individuals in mDC but not in the MDSC population (**Figure 10E**). Thus, OMS is associated with increased MFI values of exhaustion molecules and altered expression of activation markers on DC and MDSC.

## Discussion

In the present study, we investigated the impact of OMS on the systemic levels of innate and adaptive immune cells. For the innate arm, we enumerated the total and subsets of γδ T cells, MAIT cells, DC, MDSC, monocytes, and NK cells. We deciphered the frequencies of the B, T, and Tfh subsets to cover the adaptive arm. Furthermore, we determined activation and exhaustion markers of these immune cells by assessing the MFI of activation and checkpoint markers. Our study has revealed several novel findings: 1) OMS subjects are characterized by increased peripheral levels of the Tfh pool. 2) The frequencies of classical and typical memory B cell subsets and plasmablasts are significantly elevated during OMS. 3) Immunomodulatory cells such as Tregs, Bregs, and Tfr are significantly increased in OMS subjects, and 4) the expression of exhaustion molecules on immune cells is significantly elevated in the OMS group.

We have anticipated reduced levels of suppressive cells such as Tregs and Bregs in our OMS subjects as the host immune system is activated to eliminate the infection. Tregs and Bregs are immune suppressor cells that are needed for the resolution of inflammation once the infection has cleared. To our surprise, the frequencies of suppressive cells such as Tregs and Bregs were significantly higher in OMS individuals than controls. The augmented levels of Tregs in our study are in line with the report of Wu et al. in the Chinese population (37). Furthermore, in a rat model of chronic osteomyelitis, increased peripheral levels of Tregs have been observed (38). Bregs are a small subpopulation of B cells that play a crucial role in immune tolerance by producing IL-10 (39). Compared to Tregs, the role of Bregs in OMS is relatively unknown. Bregs suppress CD4^+^ T cell proliferation and IFN-γ and IL-17 production (30). We found that both CD24^high^CD38^high^ and CD24^high^CD27^+^ Bregs are significantly elevated in OMS. The increased levels of immunomodulatory cells might dampen the activation of T and other pro-inflammatory cells in OMS. Chronic low-grade inflammation caused by persistent infection has a greater likelihood of destroying the bones. On the other hand, constant augmented levels of immunomodulatory cells might facilitate the pathogen’s establishment of chronicity that might potentially affect the bones by directly infecting the bone cells. *S. aureus* infects both osteoblasts (40), and osteoclasts, resident macrophages (41). It is clear that more mechanistic studies are needed to determine whether the immunosuppression is brought on by the host for the resolution of inflammation or by microbes to establish a chronicity in the host.

Tfh cells are specialized CD4^+^ T cells that are differentiated from naive CD4^+^ T cells. Tfh cells are mainly localized in the germinal centers (GC) of the secondary lymphoid organs. The expression of CXCR5 on Tfh facilitates the migration of Tfh into GC, which enables the interaction of Tfh with B cells. Tfh cells play an important role in the maturation, activation, and antibody production of B cells (42). Thus, these cells are mainly studied in vaccine research. Based on the expression of surface markers and cytokine production, Tfh cells are further subdivided into Tfh1, Tfh2, Tfh17, and Tfr. Tfh2 and Tfh17 are effective in aiding B cells to produce antibodies via IL-21 production (43, 44). Elevated levels of these cells have been reported in many autoimmune diseases as they facilitate the production of autoantibodies by B cells (45). Vaccine research suggests that Tfh1 cells are not efficient at helping B cells (46), whereas these cells are increased in low-grade B-cell non-Hodgkin’s lymphoma, a condition in which the body produces abnormal B cells (47). In GC, Tfr cells act as a negative regulator of Tfh-B cell interactions. Thus, improper activation of Tfr leads to excessive activation of B cells in the GC center. Hence, Tfh and Tfr balance is critical for proper B cell function, and this balance is disrupted in several autoimmune diseases (44). In contrast to steady state and autoimmune diseases, we have observed increased levels of total Tfh frequencies and their subtypes, including Tfr. The mechanistic role of Tfh in OMS is still an enigma. *S. aureus*-induced skin infection in mice increases total Tfh levels in the lymph nodes, the secondary lymphoid organ (48). But this study did not investigate the different subsets of Tfh. This augmentation of Tfh and Tfr levels is a distinct feature of OMS. This could serve as a rapid and potential biomarker for OMS screening in community and clinical settings where immediate intervention is of paramount importance. However, this hypothesis has to be confirmed with larger cohorts. The increased levels of counter-regulatory immune molecules such as Th1 (IFN-γ) and Th2 (IL-4) cytokines have been shown in other diseases (49). Nevertheless, to our knowledge, we are the first to report the increased levels of Tfh subsets in OMS.

Humoral immunity that lasts for a long time depends on memory B cells. Classical memory B cells, often referred to as “resting memory B cells”, can multiply and develop into cells that produce antibodies. They can live for months to years (50, 51). Activated memory B cells are prepared to differentiate into plasma cells that secrete antibodies (52). Atypical memory B cells are anergic or exhausted B cells that develop as a result of persistent antigenic stimulation (27, 53). It is very well documented that Tfh promotes the formation of memory B cells from naive B cells (42). The association we have seen in this study between memory B cell subsets and Tfh suggests that Tfh cells facilitate the generation of memory B cells against the bacterial antigens in the OMS. However, the expression of exhaustion molecules on these cells might make them anergic. *S. aureus* exploits B cells through the secretion of staphylococcal protein A (SpA), a cell wall protein of the bacteria. SpA associates with the Fcγ and Fab domains of specific antibodies (54–56) and blocks antibody-mediated phagocytosis while simultaneously causing proliferative B cell apoptosis. This allows the pathogen to influence B cell fate and function (4). When particular memory B cells are exposed to the same antigen repeatedly, they are quickly reactivated, which may potentially engage fresh GC reactions and develop into transitory plasmablasts or stay in the body as memory B cells (57). Plasmablasts with short half-lives occur in the circulation briefly, and their frequency can be very high. The chronic nature of infection in OMS subjects might allow the memory B cells to constantly encounter the antigen in these subjects. The positive association of plasmablast with Tfh has already been reported in IgG4-related diseases (58). Plasmablasts also facilitate the differentiation of naive T cells into Tfh via IL-6 (59). However, SpA-specific antibodies from circulating plasmablasts and plasma cells are frequently ineffective against the bacteria (11, 60). Hence, plasmablast and Tfh may be assisting each other in the OMS condition, but whether these cells confer protection against the bacteria in OMS needs to be investigated.

Checkpoint molecules are regulators of the immune system that are expressed on immune cells. These molecules both positively and negatively regulating immune checkpoint molecules exist in the system. In this study, we have investigated the expression of PD-1 and TIM-3 and their potential ligands. PD-1 is primarily expressed on T cells, where it interacts with PD-L1/PD-L2 on antigen-presenting cells (61). This leads to the negative regulation of T cell function. TIM-3 is expressed on immune cells that bind with Gal-9, whereby it elicits its inhibitory function (62). Except for reduced TIM-3 expression on Tfh cells, OMS infection had no effect on the expression of PD-1 and TIM-3 on conventional, unconventional and specialized T cells. Furthermore, we observed increased expression of TIM-3 on B cells, NK cells, monocyte subsets, DC, and MDSC. Interestingly, checkpoint ligands, PD-L1, and Gal-9 are also upregulated in monocyte subsets, with Gal-9 upregulated in DC and MDSC. This suggests that the interaction of TIM-3/Gal-9 and PD-1/PD-L1 on the immune cells dampens the inflammation in OMS patients. Future studies are needed to investigate how bacteria regulate checkpoint signaling, whether by direct interaction with the immune cells or by secretory products. It has been demonstrated that *S. aureus* possesses several immune evading strategies, including escaping from resident macrophage activities in the bone. *S. aureus* infects osteoclasts and divides within the cells by avoiding the phagolysosome pathway (41). It has to be investigated whether checkpoint molecules have any role in this mechanism since PD-1 expression on the resident macrophages reduces phagocytosis in a tumor model (63). Li et al. recently demonstrated the impact of PD-1 in *S. aureus*-driven OMS in mice (64). In macrophages around the abscess in the *S. aureus*-infected bone, PD-1/PD-L1 expression was substantially up-regulated. PD-1/PD-L1 interaction promoted mitophagy reducing the generation of mitochondrial reactive oxygen species (ROS), which in turn reduced the ability of macrophages to eliminate bacteria. Interestingly, adjuvanting gentamicin, an antibacterial drug, with PD-L1 or PD-1 neutralizing antibodies or PD-L1 deletion significantly decreased mitophagy in bone marrow macrophages, improved bacterial clearance in bone tissue and implants, and decreased bone degeneration in mice.

T cell signaling is primarily dependent on three signals (65, 66). 1) Antigen presentation by antigen presenting cells (APC) through MHC molecules to T cell receptor (TCR) on T cells, 2) costimulation of CD28 on T cells by CD80/CD86 from APC, and 3) cytokines in the milieu. Our findings showed that OMS significantly downregulates the expression of MHCII and co-stimulatory molecules on APC, which might reduce the activation of T cells. The increased expression of negative regulators of the immune system and the reduced expression of antigen presenting molecules on the APC might dampen the activation of the immune system against the pathogen, which might eventually lead to the establishment of chronic OMS.

Our study was limited by being a cross-sectional study with a small patient cohort. Hence, it is hard to decipher the cause-and-effect mechanisms. Nevertheless, the strength of our study is that we investigated the detailed enumeration of innate and adaptive immune cells and the expression of checkpoint molecules in OMS. From our findings, it could be speculated that it is a microbial strategy to dampen the host immune system by increasing the expression of exhaustion molecules on immune cells and skewing the immune system towards an anti-inflammatory phenotype. Thereby, microbes can form a niche in the host to establish a chronic infection. Next, it is intriguing to explore how the pathogen is escaping the host immune system through the activities of immune suppressor cells (Tregs, Bregs and Tfr), immune exhaustion via checkpoint molecules, and interference with antigen presentation by dampening the expression of antigen presenting molecules. Additionally, future research should examine whether bacteria are eliciting these effects by direct interaction with host immune cells or through an alternative pathway. Overall, chronic OMS is characterized by increased frequencies of the Tfh family and increased expression of TIM-3 and its ligands on immune cells. However, further studies in a larger cohort and mechanistic studies would be helpful to develop immune biomarkers and immunotherapeutic approaches for chronic OMS subjects, especially those who have antibiotic resistance. These biomarkers would also be helpful in predicting the progression and recurrence of the disease and the treatment outcome.

## Data availability statement

The raw data supporting the conclusions of this article will be made available by the authors, without undue reservation.

## Ethics statement

The studies involving humans were approved by the local ethics committee of the University Hospital Bonn. The studies were conducted in accordance with the local legislation and institutional requirements. The participants provided their written informed consent to participate in this study.

## Author contributions

JS: Conceptualization, Data curation, Formal analysis, Investigation, Methodology, Visualization, Writing – original draft, Writing – review & editing. RH: Conceptualization, Formal analysis, Investigation, Writing – review & editing, Methodology, Resources. FS: Formal analysis, Investigation, Methodology, Writing – review & editing. RO: Formal analysis, Investigation, Methodology, Writing – review & editing. KW: Formal analysis, Investigation, Methodology, Resources, Writing – review & editing. BS: Formal analysis, Investigation, Methodology, Resources, Writing – review & editing. PS: Formal analysis, Investigation, Methodology, Writing – review & editing. CB: Formal analysis, Methodology, Writing – review & editing, Resources. DW: Formal analysis, Methodology, Writing – review & editing, Resources. AS: Formal analysis, Investigation, Methodology, Writing – review & editing, Resources. FS: Conceptualization, Formal analysis, Funding acquisition, Investigation, Methodology, Supervision, Writing – original draft, Writing – review & editing, Resources.

## Supplementary material

Supplementary Figure 1 Elevated CD25^+^ conventional, specialized T cells and unconventional T cells in OMS subjects. Frequencies of CD25^+^ **(A)** conventional (CD4^+^ and CD8^+^) T cells. Frequencies of CD25^+^ **(B)** specialized (Tfh and γδ) T cells and **(C)** unconventional (MAIT) T cells. The bars represent the mean ± SD. Statistical significance was determined by student t or Mann-Whitney U test and *p<0.05, **p<0.01 and ***p<0.001.
