# Supplementary figures and images for: Osteomyelitis is associated with increased anti-inflammatory response and immune exhaustion
Source: Front Immunol. 2024 Apr 26;15:1396592. doi: 10.3389/fimmu.2024.1396592 (PMC11082283; doi:10.3389/fimmu.2024.1396592)

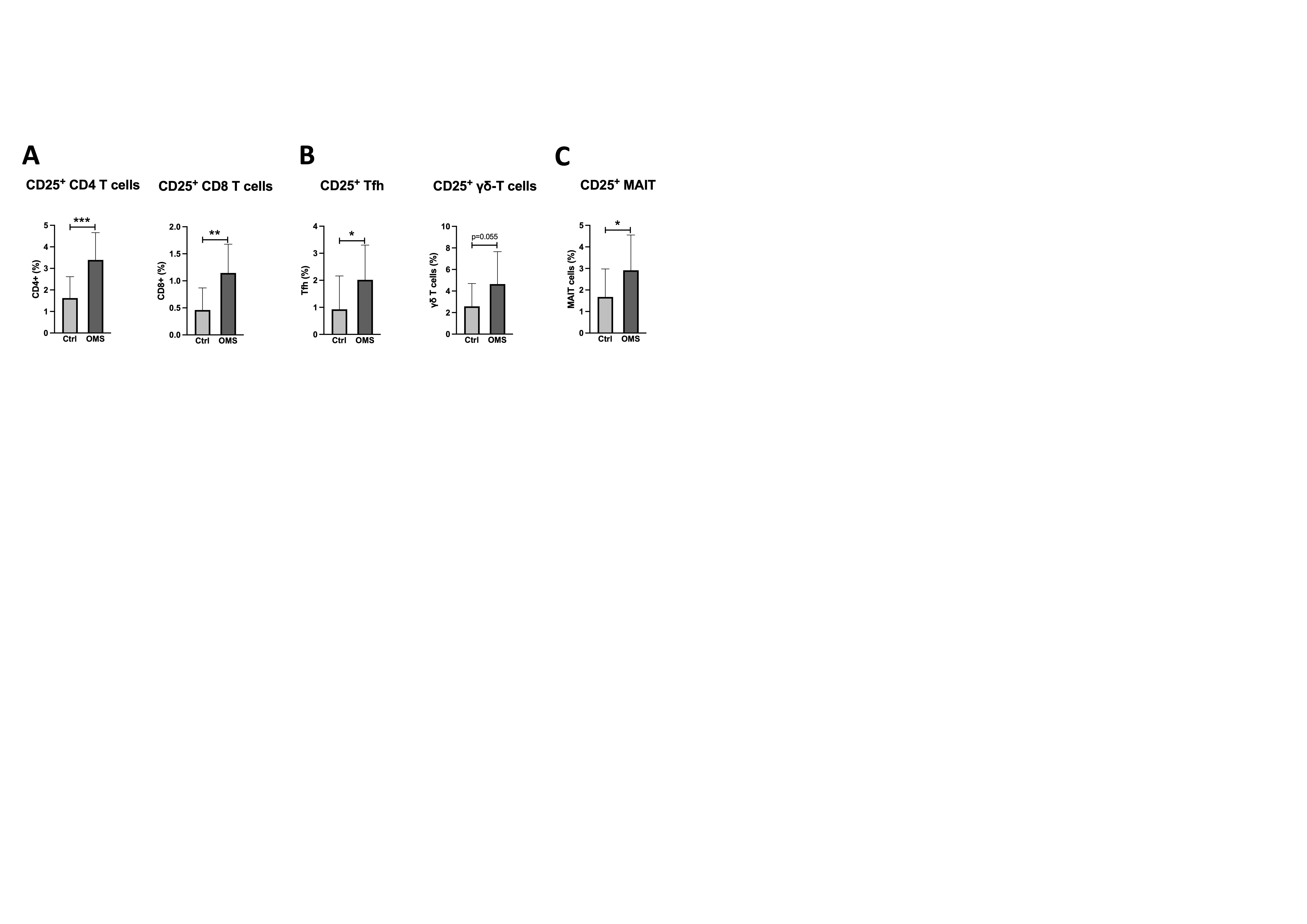

Supplement: Supplementary Figure 1 — Elevated CD25+ conventional, specialized T cells and unconventional T cells in OMS subjects. Frequencies of CD25+ (A) conventional (CD4+ and CD8+) T cells. Frequencies of CD25+ (B) specialized (Tfh and γδ) T cells and (C) unconventional (MAIT) T cells. The bars represent the mean ± SD. Statistical significance was determined by student t or Mann-Whitney U test and *p<0.05, **p<0.01 and ***p<0.001. [file Image_1.jpg]
